# Intensive Conventional Rehabilitation for Reducing Upper Limb Co-contraction and Functional Improvement in Parkinson’s Disease: A Case Report

**DOI:** 10.7759/cureus.100647

**Published:** 2026-01-02

**Authors:** Bando Kyota, Terunori Sano, Yuta Miyazaki, Masaki Takao, Takatoshi Hara

**Affiliations:** 1 Department of Rehabilitation, National Center Hospital, National Center of Neurology and Psychiatry, Tokyo, JPN; 2 Department of General Internal and Laboratory Medicine, National Center of Neurology and Psychiatry, Tokyo, JPN

**Keywords:** case report, co-contraction, electromyography, parkinson’s disease, rehabilitation, upper limb function

## Abstract

Upper limb dysfunction significantly impairs activities of daily living in patients with Parkinson’s disease (PD). This dysfunction is partially attributable to abnormal muscle contraction. Although robot-assisted therapy improves upper limb function in patients with PD, these devices are expensive and not easily accessible in standard rehabilitation facilities. Non-robotic interventions, such as repetitive large-amplitude movements, improve upper-limb performance; however, whether these conventional exercises could improve co-contraction remains unclear. Herein, we present the case of a 70-year-old man with PD (Hoehn and Yahr Scale, stage 2) who presented with bradykinesia and difficulty moving his right arm. He underwent a two-week intensive rehabilitation program (1 h per day for five days a week) focusing on upper limb training. Following the intervention, the nine-hole peg test times and mean co-contraction index during reaching showed clinically significant improvements in both upper limbs, exceeding the minimal detectable change. This functional recovery was accompanied by a physiological reduction in muscle co-contraction. These results suggest that intensive conventional rehabilitation could be an effective and accessible alternative to robotic therapy for modulating muscle tone.

## Introduction

Parkinson’s disease (PD) is a progressive neurodegenerative disease. PD-associated decline in upper limb function is a major inhibitory factor for activities of daily living [1]. In PD, pathophysiological upper limb dysfunction is influenced by abnormal muscle co-contraction, which contributes to rigidity and inefficient movement patterns [2]. Muscle co-contraction is defined as the simultaneous activation of agonist and antagonist muscles around a joint [3]. Muscle co-contraction is a necessary physiological mechanism for joint stability and precision in healthy individuals; whereas, pathological co-contraction in patients with PD is characterized by excessive and prolonged activity. This results in increased joint stiffness and resistance to movement, which clinically manifests as rigidity and hinders the execution of smooth, large-amplitude motions. Recently reported evidence indicates that robot-assisted therapy can effectively improve upper-limb motor function in patients with PD [4]. However, research into the physiological mechanisms, such as changes in muscle co-contraction, that mediate these improvements remains limited compared with the evaluation of the clinical outcomes. Furthermore, robotic devices are expensive and cannot easily be used in ordinary rehabilitation facilities. In contrast, non-robotic interventions, using tools such as repetitive movements and exercises with large amplitudes, effectively improve upper limb function in PD [5]. Although these exercises improve upper limb performance and confer clinical benefits, it remains unclear whether repetitive exercises or large-amplitude movements lead to physiological improvements in co-contractions. In this case report, we present an intervention that was aimed at improving upper limb function and resulted in functional recovery as well as a reduction in co-contraction.

## Case presentation

History and clinical findings

A 70-year-old man (height: 172 cm, weight: 60 kg), diagnosed with PD (Hoehn and Yahr (HY) stage 2, indicating bilateral symptoms without postural instability), developed bradykinesia at approximately 66 years of age. The patient had an unremarkable medical history without showing other neurological or musculoskeletal disorders. By 67 years of age, he began experiencing difficulty in moving his right upper limb and developed a resting tremor and dropped head syndrome. The patient was admitted to our hospital for rehabilitation. His primary complaint was that compared with the left arm, the right arm was difficult to move, and movements were slow.

**Figure 1:**
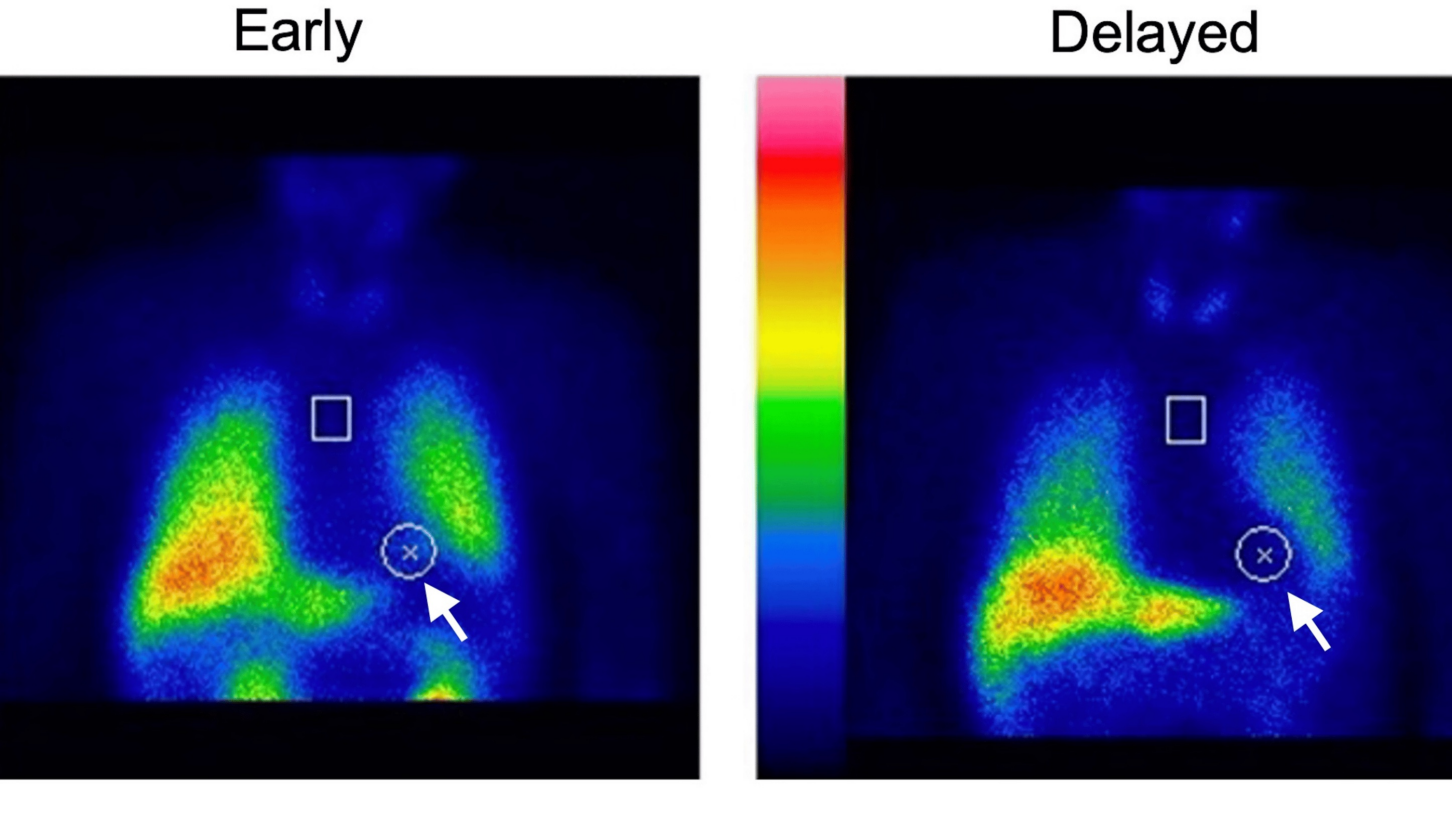
123I-MIBG myocardial scintigraphy The image shows reduced accumulation in the heart (white arrows), with heart-to-mediastinum (H/M) ratios of 1.84 (early) and 1.52 (delayed), and a washout rate of 43.4%. 123-I MIBG: 123 Iodine-metaiodobenzylguanidine.

**Figure 2:**
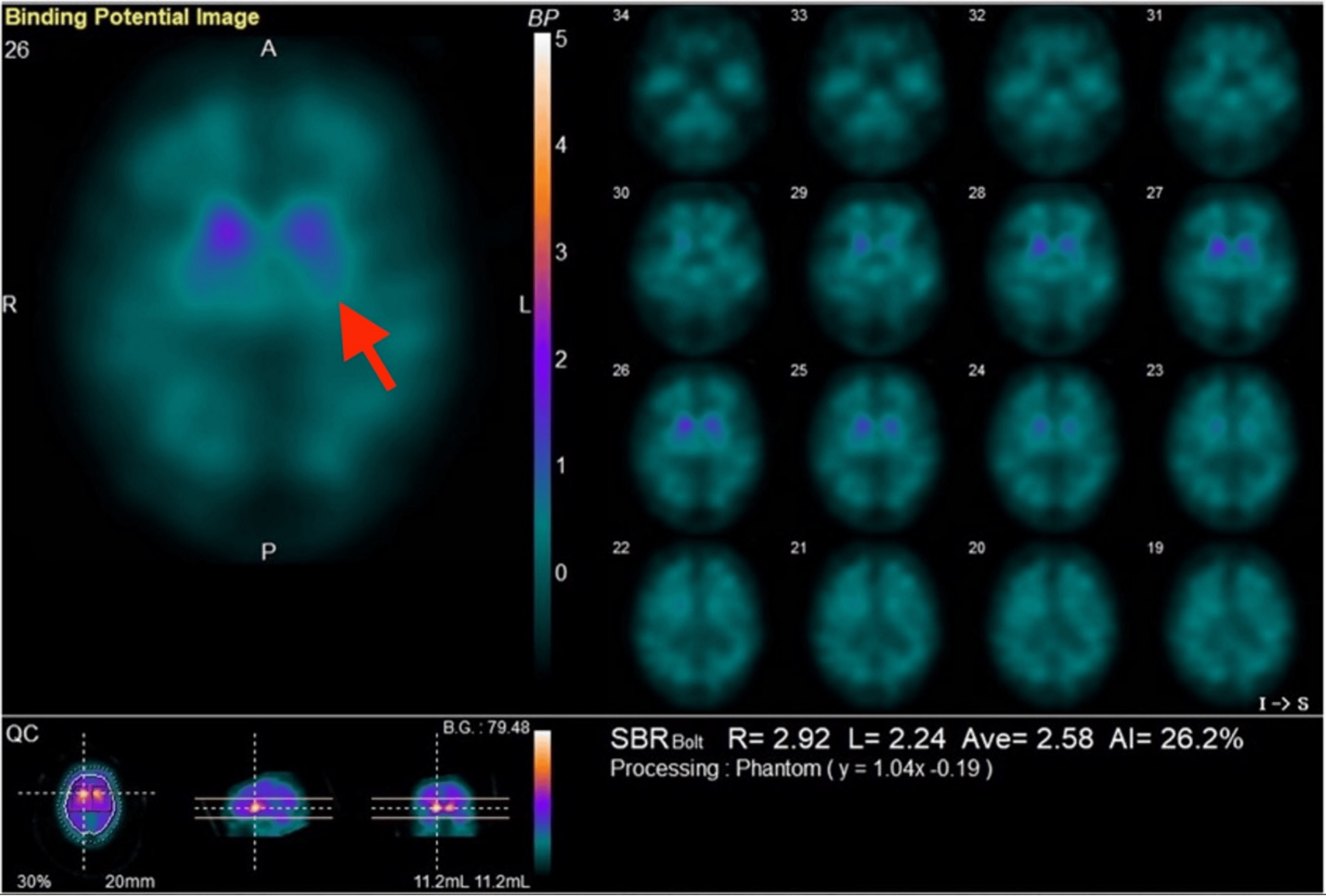
Dopamine transporter SPECT (DaTscan) The scan demonstrates reduced specific binding ratios (SBR) in the striatum, particularly in the left basal ganglia (red arrows), consistent with the patient's right-sided motor symptoms. SPECT: Single-photon emission computed tomography.

Diagnostic assessment: metaiodobenzylguanidine myocardial scintigraphy was performed to support the clinical diagnosis of PD by evaluating cardiac sympathetic denervation. The scan revealed heart-to-mediastinum ratios of 1.84 and 1.52 in the early and delayed phases, respectively, with a washout rate of 43.4% (Figure 1). Additionally, a dopamine transporter single-photon emission computed tomography, conducted on April 10, 2025, showed specific binding ratios of 2.92 and 2.24 on the right and left sides, respectively (average = 2.58). The scan demonstrated a marked reduction in accumulation in the left basal ganglia, which correlated clinically with the right-sided motor symptoms (Figure 2).

Medication

The patient was treated with levodopa/carbidopa at a dose of 600 mg/day and pramipexole at a dose of 0.5 mg/day. This medication regimen had been stable for at least three months prior to admission; moreover, there was no change during the rehabilitation intervention period.

Therapeutic intervention

**Figure 3:**
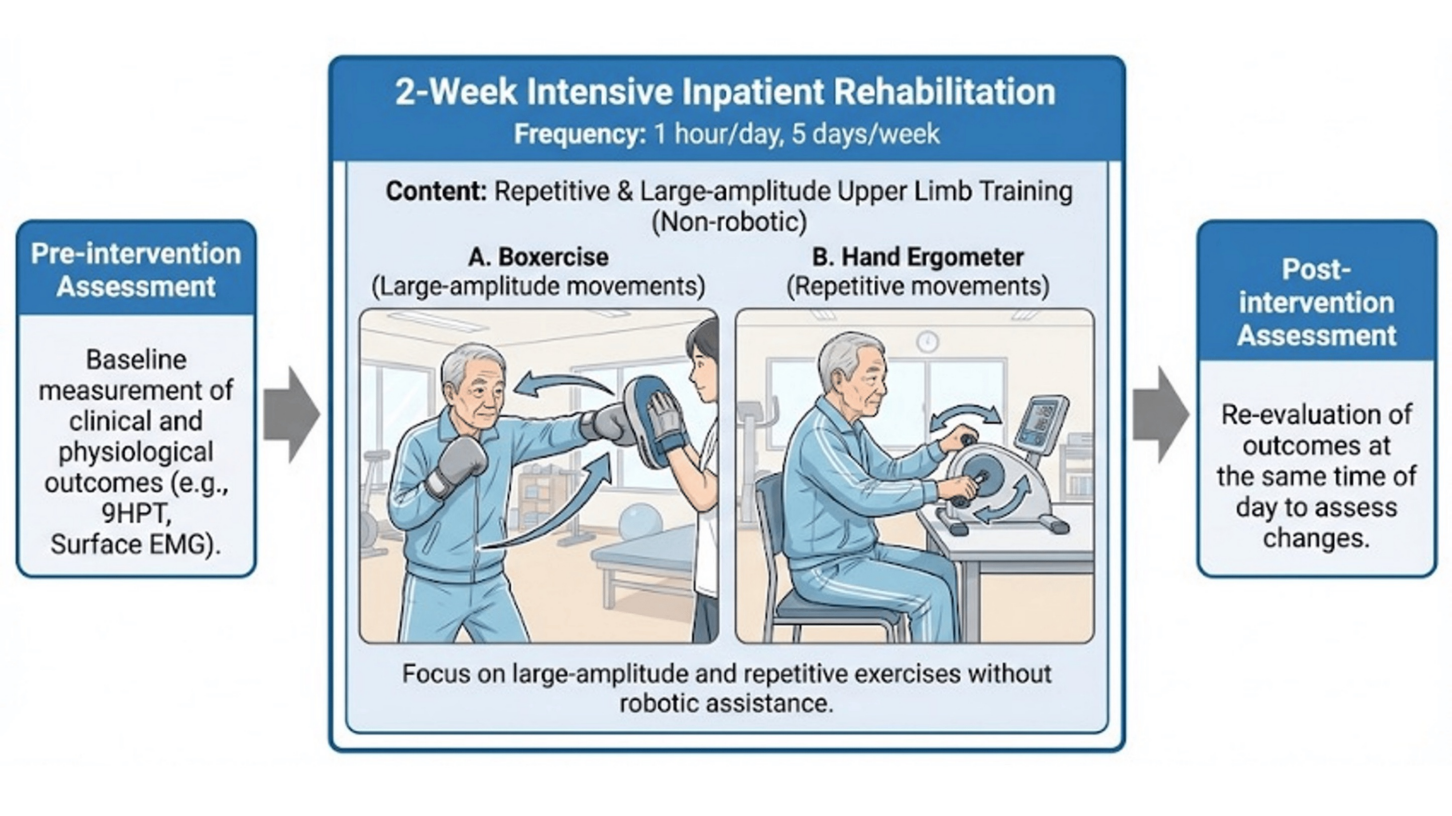
Timeline and components of the intensive conventional rehabilitation intervention The flowchart illustrates a two-week intensive inpatient rehabilitation program focused on upper limb training. The intervention was conducted for 1 h per day, five days per week. The visual elements depict examples of conventional non-robotic exercises that emphasize repetitive, large-amplitude movements, such as boxercise and hand ergometer training. Outcomes were measured both before and after the intervention to assess functional and physiological changes. Image credits: Kyota Bando, created for this manuscript.

The intervention comprised 1 h of upper-limb training per day, performed five times a week for two weeks (Figure 3). The training focused on repetitive movements and large-amplitude exercises, without using robotic devices. These specific non-robotic treatments were chosen because large-amplitude training is designed to recalibrate the patient's internal scaling of movement, which is fundamentally impaired in patients with PD [6]. Furthermore, we selected these conventional exercises over robot-assisted therapy to evaluate a treatment protocol that is highly accessible and cost-effective, making it applicable in standard rehabilitation facilities where advanced robotic technology might not be available. On the second day of the intervention, the patient reported upper limb muscle pain. However, the pain was tolerable; the intervention was continued without interruption. No other adverse events were observed during rehabilitation.

Outcome measures

The outcomes were measured both pre- and post-intervention at the same time of the day to exclude the influence of PD pharmacotherapy. The primary outcome measures included the 9-hole peg test (9HPT) for manual dexterity and patient-reported subjective improvements [7]. The 9HPT is a standardized quantitative assessment of finger dexterity, in which the patient is timed as they place nine pegs into a board and then remove them as quickly as possible. In this study, the test was performed twice per hand; the faster time (in seconds) was recorded for analysis. As a secondary outcome, we measured muscle activity during reaching movements to assess physiological changes. Surface electromyography (EMG; Delsys Inc.) was used for measuring muscle activity from the biceps brachii (BC) and triceps brachii (TC). The pre-intervention EMG assessment was conducted one day prior to starting the rehabilitation program, while the post-intervention assessment was performed on the day following the completion of the two-week intervention. During these sessions, the patient performed 10 repetitions of maximum velocity-reaching movements. The EMG signals were sampled at 1000 Hz. After bandpass filtering, the co-contraction index (CCI) was calculated (range: 0-1) to quantify the level of antagonistic muscle stiffness as follows [3]

$CCI(t) = \frac{2 \times mi(EMG_{BC}(t), EMG_{TC}(t))}{EMG_{BC}(t) + EMG_{TC}(t)}$

A value closer to 1 indicated a stronger co-contraction ratio between the agonist and antagonist muscles. The mean CCI that was observed during the reaching movement was included in the analysis.

Primary Outcomes

In the 9HPT, the time for the right upper limb decreased from 43.7 s before the intervention to 25.9 s after the intervention. For the left upper limb, it decreased from 26.3 to 19.3 s (Table 1). The minimal detectable change (MDC) has been defined as 2.6 s for the dominant hand and 1.3 s for the non-dominant hand [8]. In this patient, changes exceeding the MDC were observed bilaterally. Additionally, regarding the patient's subjective impression, there was one comment, "It has become easier to raise the left and right arms.”

**Table 1:** Changes in clinical and physiological outcomes pre- and post-Intervention Values for 9-Hole Peg Test are presented in seconds. Values for Co-contraction Index (CCI) are presented as a ratio (range 0–1), where higher values indicate stronger co-contraction. CCI values are shown as mean (95% CI).

Outcome measure		Pre-intervention	Post-intervention	Change
9-Hole Peg Test (seconds)	Right	43.7	25.9	-17.8
	Left	26.3	19.3	-7
Co-contraction Index (ratio)	Right	0.82 (0.823–0.825)	0.44 (0.439–0.444)	-0.38
	Left	0.58 (0.574–0.576)	0.43 (0.427–0.433)	-0.15

Secondary Outcomes

**Figure 4:**
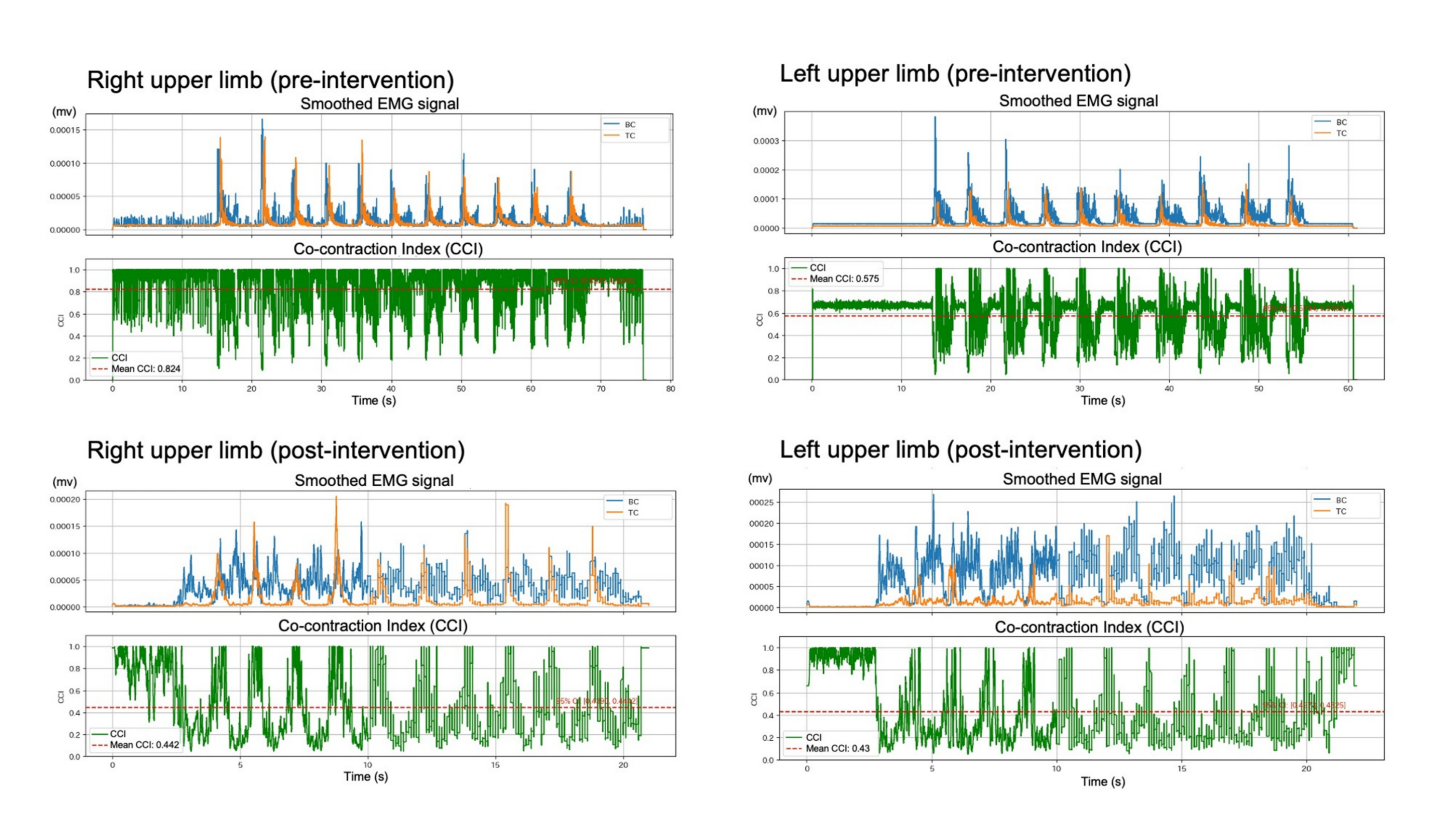
Changes in surface EMG and CCI during reaching movements This figure presents the physiological data of the BC and TC muscles during the maximum velocity reaching tasks. The top panels show the pre-intervention status of the right and left upper limbs, whereas the bottom panels show the post-intervention status. In each panel, the upper graph shows the smoothed EMG signals (full-wave rectified and 50-ms moving average) for the BC (blue line) and TC (orange line). The lower graph in each panel represents the time-varying CCI (green line), and the red dashed line indicates the mean CCI. A reduction in the overlap of agonist and antagonist muscle activities and a decrease in the mean CCI were observed following the intervention. BC: Biceps brachii, TC: Triceps brachii, CCI: Co-contraction index, EMG: Electromyography.

The mean CCI during the reaching movement improved from 0.82 (95% CI [0.823, 0.825]) before the intervention to 0.44 (95% CI [0.439, 0.444]) after the intervention in the right upper limb. In the left upper limb, the CCI improved from 0.58 (95% CI [0.574, 0.576]) to 0.43 (95% CI [0.427, 0.433]) (Table 1). Representative surface EMG waveforms and temporal changes in the CCI during the reaching tasks are shown in Figure 4.

## Discussion

This case report suggests that intensive conventional rehabilitation may improve upper limb function and reduce abnormal muscle contractions in this patient with PD. Although robotic therapy improves upper limb motor function, our results provide a hypothesis-generating observation that nonrobotic interventions, specifically repetitive and large-amplitude training, could potentially yield similar physiological benefits. The reduction in CCI (Right: 0.82 to 0.44; Left: 0.58 to 0.43) implies a reduction in the stiffness of antagonistic muscles and thereby allowing smoother movement trajectories. Our findings align with those in previous studies on large-amplitude training, such as Lee Silverman Voice Treatment BIG®, an intensive exercise program specifically designed for Parkinson’s disease that focuses on high-amplitude movements [4,8]. Although those studies primarily focused on kinematic outcomes, our case provides physiological evidence indicating that these clinical improvements may be underpinned by a reduction in antagonistic muscle stiffness. Large-amplitude movements are believed to recalibrate the sensorimotor system [6]. Enhanced sensory input may normalize the gain of motor command in the basal ganglia and thereby reduce the excessive co-contraction that is frequently observed in PD rigidity. This physiological change likely contributed to the significant functional improvements observed in the 9HPT that exceeded the MDC [8]. A potential limitation of this report is the possibility of a learning effect associated with the 9HPT. However, the concurrent reduction in the CCI suggests that the observed functional gains were not merely attributable to task familiarization, but rather, were associated with physiological changes in neuromuscular control.

## Conclusions

This case suggests that an intensive two-week conventional rehabilitation program focusing on repetitive and large-amplitude movements was associated with improved upper limb function and reduced abnormal muscle co-contraction in a patient with PD. The concurrent improvement in the 9HPT and reduction in the CCI suggest that the observed functional gains were associated with physiological changes in neuromuscular control. Crucially, these findings suggest the potential for antagonistic muscle stiffness modulation through non-robotic interventions, indicating that it may not be exclusive to robot-assisted therapy.

Clinically, these results are significant because they suggest that well-structured conventional therapy could potentially serve as a viable and accessible alternative to expensive robotic devices. Incorporating high-intensity, large-amplitude exercises, clinicians might be able to effectively manage upper limb dysfunction and rigidity, even in settings where advanced technology is unavailable. Although this is a single case report, the positive outcomes suggest the need for further larger-scale studies to explore these physiological mechanisms and establish standardized protocols for intensive non-robotic rehabilitation for PD.
